# The Effect of Adhesive Systems on Shade Matching of Composite Veneer

**DOI:** 10.3390/dj14020085

**Published:** 2026-02-03

**Authors:** Fadak Al Marar, Raghad Aljarboua, Fatimah M. Alatiyyah, Shahad AlGhamdi, Faraz Ahmed Farooqi, Lama Almuhanna, Rasha AlSheikh, Abdul Samad Khan

**Affiliations:** 1 College of Dentistry, Imam Abdulrahman Bin Faisal University, P.O. Box 1982, Dammam 31441, Saudi Arabia; fadakalmarar@gmail.com (F.A.M.); sh.alghamdi324@gmail.com (S.A.); 2 Fellowship in Orthodontics, Department of Preventive Dental Science, College of Dentistry, Imam Abdulrahman Bin Faisal University, P.O. Box 1982, Dammam 31441, Saudi Arabia; raghadaljarboua@gmail.com; 3 Department of Genetic Research, Institute for Research and Medical Consultations (IRMC), Imam Abdulrahman Bin Faisal University, P.O. Box 1982, Dammam 31441, Saudi Arabia; 4 Department of Dental Education, College of Dentistry, Imam Abdulrahman Bin Faisal University, P.O. Box 1982, Dammam 31441, Saudi Arabia; fafarooqi@iau.edu.sa; 5 Department of Restorative Dental Sciences, College of Dentistry, Imam Abdulrahman Bin Faisal University, P.O. Box 1982, Dammam 31441, Saudi Arabia; lmaalmuhanna@iau.edu.sa (L.A.); ralsheikh@iau.edu.sa (R.A.)

**Keywords:** bonding agents, beverages, composite veneers, color stability, bioactive materials

## Abstract

Objective: This study aimed to assess the impact of different adhesive systems on the color stability of composite veneers following their exposure to various common beverages. Materials and Methods: A single layer of commercially available adhesives (4th and 7th generations) and two experimental adhesives based on hydroxyapatite and bioactive glass were applied, followed by composite restoration on incisor typodonts. The typodonts were prepared with depths of 0.3, 0.5, and 0.7 mm at the cervical, middle, and incisal regions, respectively. Samples from each group were immersed in coffee, Cola, and deionized water, and color stability was analyzed on days 1 and 60. One-way and two-way analyses of variance were performed. Results: The interaction between groups and solutions was statistically significant (*p* = 0.001) across all tooth regions. Coffee and Cola caused significant color changes (*p* = 0.001). The 4th generation demonstrated better color stability than the 7th generation in the middle and cervical regions (*p*-values = 0.083 and 0.003, respectively). The findings showed that the bioactive glass-based bonding agent exhibited greater discoloration than the hydroxyapatite-based adhesive (*p* = 0.001). Conclusions: The composite thicknesses are influenced differently by adhesives with respect to shade matching. Bioactive materials-based adhesives showed more resistance towards color change than commercial adhesives.

## 1. Introduction

Aesthetic dentistry has become a primary concern for both dentists and patients, influencing the dental field to seek more convenient and beneficial treatments [1]. Currently, advances in science have significantly reduced the use of amalgam alloy and largely replaced it with composite materials. The composite offers valuable functional traits that surpass those of amalgam, thereby enhancing its applications [2]. In the field of restorative dentistry, dental resin adhesives are a fundamental component, representing a significant shift from conventional mechanical retention methods to modern adhesive techniques that facilitate both micromechanical and chemical bonding with dentin and enamel [3].

The purpose of developing adhesive systems is to increase the bond duration of dental composites to dental tissue. Moreover, from no-etch to total-etch (4th and 5th generations) to self-etch (6th and 7th generations), adhesives have undergone significant improvements [4]. The total removal of the smear layer was first introduced by the 4th-generation adhesive, which is considered the gold standard [4,5]. The main components of the 4th generation are the etchant, primer, and bonding agents, which are applied sequentially. Enamel and dentin are both etched at the same time with phosphoric acid (H_3_PO_3_) [1,4]. After rinsing them with water, the primer was applied. This will infiltrate the collagen network, forming a hybrid layer. This process will increase the bonding strength and improve dentin seal [1]. This system is considered a sensitive and time-consuming technique. This generation exhibits low-to-moderate dentin bond strength, reduced marginal leakage, and good long-term clinical performance [6].

The 5th generation is a one-bottle system that combines the primer and adhesive in a single bottle. The 6th generation bonding system is commonly referred to as a “self-etching primer.” It sought to eliminate the etching step, where they provide two bottles of primer and adhesive mixed together and applied immediately into the tooth structure [4]. This system provides sufficient dentin-bonding strength but less effective bonding to the enamel surface. The 7th-generation adhesive system is a one-bottle, self-etching agent that combines an etchant, primer, and adhesive [7]. A comparative study showed that 7th-generation adhesives performed better than 5th and 6th-generation adhesives in terms of retention [8,9]. The 4th-generation bonding agents are recommended when unprepared enamel is present, and 7th-generation bonding agents offer strong bonds and ease of use when enamel and dentin have been prepared [4,6].

Recent developments in adhesive dentistry [9] have focused on incorporating bioactive molecules into adhesive materials to enhance their biological performance and durability [5,10,11]. These molecules include amorphous calcium phosphate (ACP), a highly soluble form of calcium phosphate that can release calcium and phosphate ions to promote remineralization of demineralized dentin [5]. Hydroxyapatite (HA), the primary mineral component of natural tooth structure, can reinforce the hybrid layer and improve bond stability [10]. Bioactive glass (BG) is a silica-based material that forms a hydroxycarbonate apatite layer upon contact with physiological fluids, thereby supporting remineralization and sealing micro-gaps [11]. By integrating these bioactive agents, adhesive systems can not only enhance bioactivity—the material’s ability to interact beneficially with surrounding tissues—but also inhibit endogenous enzymatic degradation of the collagen matrix within dentin, ultimately contributing to the long-term stability and longevity of resin–dentin bonds [9]. Despite these advancements, no commercial adhesive currently contains bioactive materials. Previous studies [10,11] have found that adding nano-hydroxyapatite and nano-bioactive glass particles to the adhesive resin improved the mechanical properties of the adhesive, as well as the strength and structure of the tooth, and can prevent long-term deterioration in bond strength of a self-etch adhesive.

In dentistry, a direct composite veneer is a minimally invasive restorative procedure that involves applying a tooth-colored composite resin directly to the tooth’s facial surface to improve its color and shape [12]. The adhesive system, physical properties, thickness of the composite resin, and translucency are the main factors that influence the treatment outcome [13]. Aesthetic restoration has been observed to encounter many obstacles in imitating natural dentition. Color and optical properties remain a significant issue, as the resin matrix, photo-initiation system, type of filler particle, thickness of restoration, surface degradation, and bond interface all influence it [14,15,16].

Color stability is a critical determinant of the long-term success of composite resin restorations. In anterior teeth, the primary reason for replacing composite restorations is color alteration due to intrinsic discoloration, which is attributed to the chemical stability of the composite material. In contrast, extrinsic discoloration is correlated with factors such as food, smoking, and hygiene practices [17,18]. Age-related factors include variations in humidity and temperature, as well as stains on resin-based materials caused by exposure to solutions such as coffee, Cola, and other beverages.

The literature [19,20] presents comparative studies of different adhesive generations regarding bond strength and retention; however, limited information is available on color changes in the composite shade after placement of commercial and experimental adhesives containing bioactive materials. Therefore, this study aims to evaluate the effect of different adhesive systems (commercial and experimental) on shade matching of direct composite veneers after placement in various beverages. The color changes were examined in the composites, i.e., incisal, middle, and cervical. The null hypothesis of the study was that the type of adhesive system would not have a significant effect on the shade stability of direct composite veneers when exposed to different beverages, in the incisal, middle, and cervical regions of the composites.

## 2. Materials and Methods

**Figure 1:**
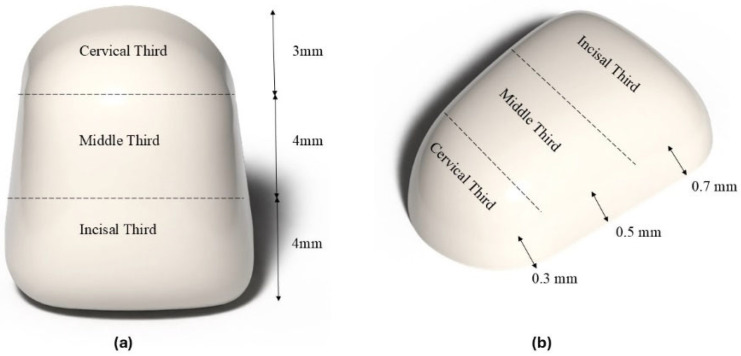
The schematic pattern of distribution of (**a**) regions and (**b**) depth of preparation on the typodont tooth.

**Figure 2:**
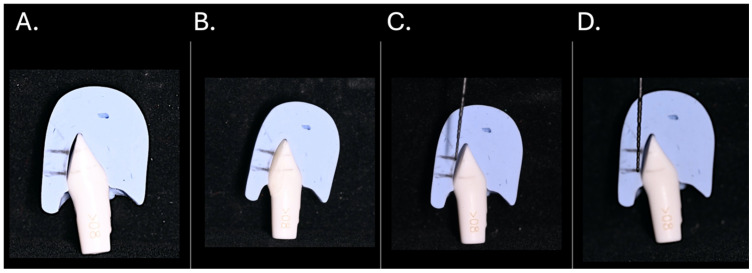
Photograph of sample preparation. (**A**) Facial tooth preparation (**B**) placement of a composite restoration precisely adapted to the putty index. Measurement of each facial tooth third, (**C**) 4 mm incisal third (**D**) 4 mm middle third.

The sample size was calculated with power analysis, whereby the investigation was carried out at a 5% significance threshold, 80% power, and 5% marginal error to have a total number of (N = 45) samples, distributed among five groups. Central incisors Typodont (ModuPRO M300, Acadental, Inc., Overland Park, KS, USA) were prepared facially using round-end tapered red Diamond bur (BluWhite Diamond Burs, Braeside VIC 3195, Melbourne, Australia). Each sample was prepared as a three-planar reduction with depths of 0.3, 0.5, and 0.7 mm at the cervical, middle, and incisal regions, respectively, as shown in Figure 1. To allow enamel adaptation and avoid a bulky appearance, preparation is limited to a light enamel reduction of approximately 0.3–0.7 mm [12]. The depth of preparation was confirmed using the putty index and the UNC-15 periodontal probe as illustrated in Figure 2.

The samples were divided into five groups based on types of adhesives, as shown in Table 1. A single coating of all adhesive groups was applied to the prepared surface using a disposable micro-brush and a stream of air for 5 s, until the solvent had evaporated. Subsequently, light curing was performed for 20 s using high-intensity blue light (LEDition, Schaan, Liechtenstein, Austria).

For the control group (CG), no adhesive was applied, and direct A2 shade enamel nano-hybrid composite (IPS Empress Direct, Ivoclar Vivadent, Schaan, Liechtenstein) was applied to the facial surface using a plastic instrument. The thickness of the composite was confirmed using translucent putty (Elite Transparent: Addition Silicone, ZHERMACK, Badia Polesine, RO, Italy). The composite was light-cured as per the manufacturer’s instructions. Finishing and polishing were performed using finishing and polishing Sof-lex discs (Sof-Lex 3M ESPE, Seefeld, Germany) (thin/fine 12.7 mm). For G1 (4th generation) and G2 (7th generation), a single layer of commercial adhesives was applied and cured according to the manufacturer’s instructions. For G3 and G4, experimental adhesives were initially prepared, and the same procedure was followed as described for G1 and G2.

### 2.1. Preparation of Experimental Adhesives

The nano-hydroxyapatite (nHA) and nano-bioactive glass (nBG) were prepared as described previously [21,22]. All monomers and photoinitiators were purchased from (Sigma Aldrich inc, St. Louis, MO, USA). For each experimental adhesive, the optimized ratios of bisphenol glycol methacrylate (bis-GMA), urethane dimethacrylate (UDMA), triethylene glycol dimethacrylate (TEGDMA), and hydroxyethyl methacrylate (HEMA) were 45:25:20:10, respectively, and were converted into the corresponding weight percentages. The weight percentages of nHA and nBG were 15 wt.% for each experimental adhesive. Camphorquinone and ethyl 4-(dimethylamino) benzoate (EDBA) were used as photoinitiators, each at 0.5 wt%. Initially, all monomers were mixed for 60 min at room temperature; then, nHA and nBG were mixed separately for their respective groups and stirred for 24 h. Then, photoinitiators were mixed in the dark and stirred for 30 min. Lastly, 10 wt.% ethanol was used as the solvent and was mixed for 60 min. After mixing thoroughly, each experimental adhesive was stored in dark bottles.

### 2.2. Media Preparation and Immersion Procedure

Samples from each group were divided into media: Cola, Coffee, and deionized water. Cola (The Coca-Cola Bottling Company of Saudi Arabia, Riyadh, Saudi Arabia) was used as received and stored at refrigerated temperatures. For coffee, 5 g of coffee (Illycaffè S.p.A., Trieste TS, Italy) was added to 250 mL of water, heated to a low flame, and removed before boiling (according to the manufacturer’s instructions). For each media, pH and temperature were measured (Apera Instruments Value Series EC20 Conductivity (EC) Pocket Tester Kit), and for coffee and Cola, the pH and temperature were pH 5.1, 122 °F, and pH 2.4, 48.4 °F, respectively. Samples from each group were immersed in different media, i.e., deionized water, coffee, and Cola. For coffee and Cola, samples were immersed for 10 min daily, then washed with running water and re-stored in deionized water, whereas samples in deionized water were stored continuously. All three media were refreshed daily, and the samples were immersed in their respective media for 60 days. Because water absorption and staining persist over time, color change cannot be properly evaluated at days 7 or 30 alone. Instead, an intermediate period, such as 60 days, was chosen to understand material behavior.

### 2.3. Color Change Analysis

The color change analysis of the incisal, middle, and cervical regions of samples immersed in coffee, Cola, and deionized water was conducted on days 1 and 60. Before and after immersion, all samples were measured using a Spectrophotometer (Minolta CR-300, Minolta Co., Osaka, Japan), against a black background. The spectrophotometer was calibrated using the manufacturer’s recommendations. Three readings were taken from each sample, and the average was calculated to evaluate the effect of different media on the tested groups.

The clinical spectrophotometer’s display uses the CIE L*a*b* color scheme. L* stands for the color’s lightness or black/white quality. The color’s chromatic characteristics are defined by a* and b*. The red–green axis is represented by the a* coordinate, and the yellow–blue axis by the b* coordinate. The following formula was used to calculate the color changes:

ΔE* = [(L_1_* − L_0_*)2 + (a_1_* − a_0_*)2 + (b_1_* − b_0_*)2] ½ (1)

### 2.4. Statistical Analysis

Analysis was conducted using SPSS software (version 24, IBM Corp., Armonk, NY, USA). The normality of the data was assessed using the Shapiro–Wilk test, and *p*-values greater than 0.05, indicating that the data followed a normal distribution. Therefore, parametric tests were employed for further analysis. One-way analysis of variance (ANOVA) was used to compare the effects of different solutions (deionized water, coffee, and Cola) across tooth regions (incisal, middle, and cervical) within each group. Additionally, two-way ANOVA was performed to assess the interaction effects between solution type and tooth region within each group, as well as between solution type and experimental group across each position. Upon observing significant differences, pairwise comparisons a were performed using Tukey’s post hoc test, and superscript letters were used to identify statistically significant differences both within columns (lowercase letters) and across rows (uppercase letters). A *p*-value of less than 0.05 was considered statistically significant.

## 3. Results

The comparative ΔE values, categorized by solution type and region of teeth within each group, are outlined in Table 2. In the CG, the highest ΔE value at the incisal region was observed with coffee (11.71 ± 1.87), followed by deionized water (10.46 ± 0.69) and Cola (5.76 ± 1.39), with a statistically significant difference (*p* = 0.005). Similarly, the middle region showed a significantly higher value in the coffee group (16.38 ± 1.66) than in the deionized water group (12.67 ± 3.10) and the Cola group (4.13 ± 0.97), with a significant difference within the coffee group (*p* = 0.001). At the cervical region, a similar trend was observed, where coffee (13.53 ± 1.48) showed higher staining than deionized water (11.94 ± 2.01) and Cola (4.40 ± 0.47) (*p* = 0.001). When comparing solutions within each region, coffee consistently caused significantly higher discoloration than Cola (*p* < 0.05). However, two-way ANOVA revealed that the interaction between region and solution was not statistically significant (*p* = 0.061).

In G1, significant differences in discoloration were observed at the incisal (*p* = 0.001) and cervical (*p* = 0.003) regions. At the incisal region, Cola (7.88 ± 0.68) caused more staining compared to deionized water (5.89 ± 0.26) and coffee (2.79 ± 0.72). In contrast, in the cervical region, deionized water (15.84 ± 1.21) and coffee (14.49 ± 2.34) showed higher discoloration than Cola (4.76 ± 0.41). No statistically significant differences were observed in the middle region (*p* = 0.083). Across solutions, significant differences were observed for deionized water (*p* = 0.001) and coffee (*p* = 0.003), but not for Cola (*p* = 0.078). The interaction between position and solution was statistically significant (*p* = 0.001).

In G2, the middle (*p* = 0.010) and cervical (*p* = 0.001) regions showed statistically significant differences among the solutions. The middle region had the highest value in the coffee group (13.48 ± 2.38), followed by deionized water (7.36 ± 0.55) and Cola (5.78 ± 1.40) groups. Similar trends were observed at the cervical region, where coffee showed significantly higher value (16.64 ± 1.69) than both deionized water (3.53 ± 1.15) and Cola (7.08 ± 2.39), whereby no significant difference was observed at the incisal region (*p* = 0.269). Across solutions, significant differences were found for deionized water (*p* = 0.001) and coffee (*p* = 0.003). The interaction effect was statistically significant (*p* = 0.001).

Table 3 compares the solution type and tooth region across the experimental groups. At the incisal region of G3, Cola caused the highest discoloration (2.56 ± 0.56), followed by deionized water (2.13 ± 0.29), while coffee caused the least (0.36 ± 0.11), with a significant *p*-value of 0.001. In the middle region, coffee caused significantly higher discoloration (6.12 ± 0.34) compared to deionized water (1.52 ± 0.33) and Cola (1.46 ± 0.43) (*p* = 0.001). For the cervical region, deionized water had the highest mean (6.65 ± 0.50), followed by Cola (5.21 ± 0.34) and coffee (5.04 ± 0.08) (*p* = 0.003). Significant differences between solutions were observed at each position (*p* = 0.001). The interaction between regions and solutions was also significant (*p* = 0.001).

For G4, the incisal region showed the highest discoloration with deionized water (17.15 ± 1.53), followed by coffee (5.18 ± 0.56) and Cola (0.59 ± 0.08) (*p* = 0.001). In the middle region, coffee (11.11 ± 0.74) caused the highest change, followed by deionized water (10.44 ± 1.11) and Cola (3.63 ± 0.20) (*p* = 0.001). The cervical region also showed significant differences (*p* = 0.037), with coffee (11.03 ± 2.21) having the highest value. Significant differences were observed for all three solutions, i.e., deionized water, coffee, and Cola (*p*-values = 0.001, 0.003, and 0.015, respectively). The interaction between solution and region was significant (*p* = 0.001).

**Figure 3:**
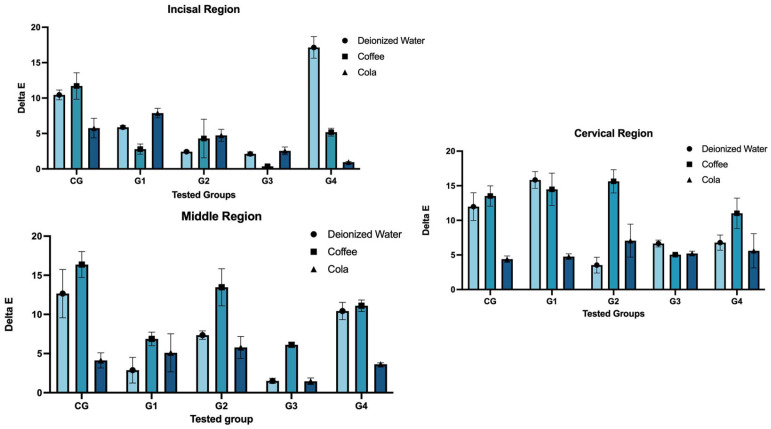
Comparison between solution type and groups within each tooth region.

Table 4 and Figure 3 present a comparison of solution types across tooth regions. At the incisal region, significant differences were observed among the groups for all solution types. Deionized water caused the highest discoloration in G4 (17.15 ± 1.53), while the lowest was observed in G3 (2.13 ± 0.29) (*p* = 0.001). Coffee showed the highest value in CG (11.71 ± 1.87) and the lowest in G3 (0.36 ± 0.11) (*p* = 0.001). For Cola, the maximum was found in G1 (7.88 ± 0.68) and the minimum in G4 (0.59 ± 0.08) (*p* = 0.001). The interaction between group and solution was statistically significant (*p* = 0.001).

In the middle region, the coffee group showed the highest discoloration in CG (16.38 ± 1.66), while the lowest was in G3 (6.12 ± 0.34) (*p* = 0.001). For deionized water, CG again had the highest score (12.67 ± 3.10), and G3 had the lowest (1.52 ± 0.33) (*p* = 0.001). Cola showed the greatest effect in G2 (5.78 ± 1.40) and the least in G3 (1.46 ± 0.43) (*p* = 0.024). The interaction between group and solution was statistically significant (*p* = 0.001).

For the cervical region, the coffee group showed the highest discoloration in G2 (16.64 ± 1.69), and the lowest in G3 (5.04 ± 0.08) (*p* = 0.001). The deionized water group had the highest value in G1 (15.84 ± 1.21) and the lowest in G2 (3.53 ± 1.15) (*p* = 0.001). For Cola, the differences between groups were not significant (*p* = 0.333), though G4 had a lower mean score (5.60 ± 2.48). The interaction between group and solution was significant (*p* = 0.001).

## 4. Discussion

The semi-translucent nature of the resin composite material, the foundation color, material thickness, and transparency all affect the final color of resin composite restorations [10,23,24]. Color stability and matching are essential for aesthetic maintenance to be clinically satisfactory. The adhesive system can be regarded as a factor influencing the color of resin composite restorations [15,23]. Thus, this study investigated how adhesive systems affected color after being submerged in various media. Overall, the null hypothesis is partially rejected due to differences in shade among groups. In this study, the total-etch 4th-generation and self-etch 7th-generation adhesives were compared with bioactive inorganic materials using experimental adhesives. One of the main ingredients of the total-etch method is phosphoric acid, whereas self-etch contains water. The proposed amount of water is insignificant, rendering the agent ineffective in terms of hydrolysis and chemical breakdown [24]. Furthermore, due to the combination of acidic etching agents in the solution, an adverse reaction occurs with the composite initiator [4].

Many variables can cause aesthetic restorative products to discolor, such as pH, titratable acidity, aging, and the penetration or absorption of food coloring, which can affect the degree of visible staining [7,25]. The current investigation showed variations in ΔE values across media and regions. The primary cause of discoloration may be water absorption in the resin matrix. Increased water sorption causes the resin to expand and become more plastic, leading to the formation of microcracks. Food and drink stains consequently seep into the interfacial spaces or microcracks between the resin and filler, resulting in discoloration [26,27]. Swelling of the polymers results in discoloration at marginal surfaces, reducing the frictional forces between polymer chains [27,28].

The presence of monomers in adhesives affects their water absorption capacity. The 4th- and 7th-generation adhesives are methacrylate-based; however, the exact composition was not available. The authors are uncertain about the presence of bis-GMA, UDMA, or other dimethacrylates. In the experimental adhesives, bis-GMA and UDMA were used at higher concentrations, which are more hydrophobic than the low-molecular-weight monomers TEGDMA and HEMA. Furthermore, Bis-GMA was selected due to its excellent performance as a base resin matrix in resin-based materials, offering several advantages over other dimethacrylate-based systems, including reduced polymerization shrinkage, adequate mechanical strength, and favorable adhesive properties [29].

In this study, samples were immersed in coffee, assuming an average daily intake of one cup of coffee for thirty minutes, a week of coffee immersion was thought to approximate a year of coffee consumption. Additionally, according to the coffee manufacturer, the average time it takes a coffee drinker to finish one cup is 15 min, and the average person consumes 3.2 cups of coffee per day [30,31]. According to these findings, if a coffee solution is continuously in the mouth while drinking, a one-week coffee immersion may mimic a seven-month coffee consumption period. Compared with 7 months of coffee consumption, a 1-week coffee immersion most likely replicated longer oral tissue contact times due to the shorter actual coffee contact time [31]. The ΔE values following immersion in coffee, regardless of position, were higher than the previously reported acceptability threshold of 5.5 [32]. Carbonic acid in Cola did not mark stains as much as coffee, despite having the lowest pH, which might harm the material’s surface integrity [33,34]. This could be because the relationship between pH and overall acidity, and the resulting color change, is not linear. Cola does not include a yellow colorant, whereas coffee includes yellow colorants that, through adsorption and absorption, discolor the materials. The polymer phase’s compatibility with the yellow colorants in coffee is most likely what allowed the colorants to absorb and permeate into the organic phase of the materials [33]. This was caused by the resin matrix softening, which dislodged filler particles and facilitated the stains’ adhesion.

In this study, darker shades were found in the cervical (0.3 mm) and middle (0.5 mm) regions compared to the incisal (0.7 mm) area. This might be justified by the fact that both restoration thicknesses have a major impact on the final color of tooth structure. This variation in thickness was determined by tooth type and anatomical location; thus, this thickness was used for all experimental setups in this investigation.

Several studies [35,36,37] indicated that the potential for color adjustment is inversely proportional to the composite’s thickness. This means thicker layers of composite are less able to blend with the natural tooth color. The universal adhesive tested showed more noticeable color changes with a more translucent resin composite, yet this was clinically acceptable [36,38].

Bioactive restorative techniques can be used to increase the longevity of resin-dentin interfaces. It has been suggested that mineral preservation of active endogenous might decrease matrix degradation [38]. Because of their exceptional capacity to release phosphate or calcium, bioactive glasses have been shown to stimulate remineralization and preserve the resin-dentin interface when incorporated into resin-based products [39]. The mineral that forms the structure of teeth is hydroxyapatite particles. It serves as a precursor to minerals and repairs those lost during demineralization. In this study, nano-structured hydroxyapatite and bioactive glass particles were used. The nanoparticle has a high surface area, which enhances interaction between the restorative materials and the tooth, thereby strengthening the bond and reducing hypersensitivity by sealing the dentinal tubules. Bioactive glass particles enhance bonding strength and remineralization and exhibit antimicrobial activity [39,40]. Previous studies have demonstrated that bioactive materials can increase ion diffusion through adhesive-bonded dentine, as simplified all-in-one bonding systems are permeable, thereby raising the mineral-to-matrix ratio and reducing permeability and nano-leakage at the resin-dentine interface [40]. Bioactive particles affect color stability by influencing water sorption, resin matrix integrity, filler-matrix interactions, and surface chemistry. The incorporation of ion-releasing fillers in bioactive materials promotes a favourable biological response. Additionally, smaller and uniformly distributed filler particles reduce interfacial gaps and water diffusion, thereby limiting hydrolytic degradation and stain absorption [41,42]. These are possible factors that led to color changes in experimental groups. Overall, the experimental groups showed that the bioactive glass-based bonding agent exhibited greater discoloration than the hydroxyapatite-based bonding agent. This could be due to the higher resorbability rate of bioactive glass. In this study, typodont teeth were used, and it is expected that the high calcium release will facilitate the formation of an apatite layer on the natural tooth structure. The formation of an apatite layer can improve the optical properties of the tooth surface. The typodont was used in this study to achieve uniformity, as variations in tooth color of extracted teeth have been observed previously due to age, gender, dietary habits, etc. [41].

The dynamic nature of the oral environment, characterized by continuous fluctuations in pH, mechanical stress, and temperature, can significantly influence the color stability of aesthetic and bioactive restorative materials. Although the present study was conducted under controlled conditions to standardize testing variables, such an environment may not fully replicate the long-term complexities encountered in clinical settings, thereby limiting the direct applicability of the findings. In addition, the color change in the composite and adhesive materials was not evaluated independently; consequently, the precise contribution of the adhesive material to the overall color change in the composite restoration could not be quantified. Future studies should assess composite and adhesive materials separately, explore alternative artificial saliva models, and incorporate clinical trials to better determine individual material effects and to validate the findings under real oral conditions.

## 5. Conclusions

Within the limitations of this study, it is concluded that the color stability of restorative materials varies significantly with the type of adhesive used and the immersion medium. Bioactive glass (G4) and hydroxyapatite-based (G3) bonding agents demonstrated greater resistance to discoloration under most immersion conditions compared to the control and commercial groups (G1 and G2). Coffee and Cola produced significantly greater color changes than deionized water, highlighting their strong staining potential. When comparing G3 and G4, color changes were comparable across most regions and beverages; however, G3 demonstrated lower color change values, particularly in the middle and cervical regions. The middle region exhibited the most pronounced color change, followed by the incisal region, while the cervical region showed the least discoloration across all bonding agents and immersion media. It is recommended to employ enhanced surface sealing methods and optimized polishing protocols. These findings indicate that bioactive glass and hydroxyapatite-based adhesive systems offer promising color stability, suggesting their potential advantage in maintaining long-term aesthetics of restorations exposed to common dietary staining agents.

## Figures and Tables

**Table 1:** Composition of the adhesives used in this study.

Groups (Labeling)	Monomers (Resin Matrix)	Fillers
Control group (CG)	No bonding was applied	
4th Generation (G1) (Optibond FL adhesive, Kerr, Orange, CA, USA	Uncured Methacrylate Ester, Monomers (50–60 wt%), Triethylene Glycol Dimethacrylate (TEGDMA) (5–10%).	Ytterbium Trifluoride (12–17 wt%) Inert mineral fillers.
7th Generation (G2) Tetric N-Bond Universal, Ivoclar Vivadent, Amherst, NY, USA	HEMA (2-hydroxyethyl methacrylate) MCAP (methacrylated carboxylic acid), and D3MA (decanediol dimethacrylate)	Highly dispersed silicon dioxide
Experimental adhesive 1 (G3)	Bisphenol glycol methacrylate (bis-GMA), urethane dimethacrylate (UDMA), triethylene glycol dimethacrylate (TEGDMA), and hydroxyethyl methacrylate (HEMA)	nano-Hydroxyapatite 15 wt.%
Experimental adhesive 2 (G4)		nano-Bioactive glass 15 wt.%

**Table 2:** The mean and SD ΔE values of different regions of the composite after placing in deionized water, coffee, and Cola for 60 days.

CG				*p*-Values
Region	Deionized Water	Coffee	Cola	
Incisal	10.45 ± 0.69 ^A^	11.711 ± 1.87 ^a,B^	5.76 ± 1.38 ^A,B^	0.005 *
Middle	12.66 ± 3.09 ^A^	16.37 ± 1.66 ^a,B^	4.12 ± 0.97 ^B^	0.001 *
Cervical	11.93 ± 2.01 ^A^	13.52 ± 1.47 ^B^	4.40 ± 0.46 ^B^	0.001 *
*p*-values	0.487	0.039 *	0.189	
**G1**				*p*-values
Region	Deionized Water	Coffee	Cola	
Incisal	5.88 ± 0.25 ^a,A^	2.79 ± 0.72 ^a,b,A^	7.88 ± 0.678 ^A^	0.001 *
Middle	2.87 ± 1.64 ^a^	6.87 ± 0.87 ^a^	5.09 ± 2.43	0.083
Cervical	15.84 ± 1.21 ^a,A^	14.48 ± 2.34 ^b,B^	4.76 ± 0.40 ^A,B^	0.003 *
*p*-values	0.001 *	0.003 *	0.078	
**G2**				*p*-values
Region	Deionized Water	Coffee	Cola	
Incisal	2.43 ± 0.15 ^a^	4.29 ± 2.72 ^a,b^	4.74 ± 0.84	0.269
Middle	7.35 ± 0.55 ^a,b,A^	13.47 ± 2.37 ^a,A,B^	5.78 ± 1.40 ^B^	0.010 *
Cervical	3.53 ± 1.14 ^b,A^	16.64 ± 1.68 ^b,A,B^	7.07 ± 2.38 ^B^	0.001 *
*p*-values	0.001 *	0.003 *	0.303	

* statistically significant at 0.05, small letters (^a,b^) showing significant difference within the solution, and capital letters (^A,B^) showing statistical significance between the solutions horizontally.

**Table 3:** Comparison between solution type and position of teeth in the experimental groups.

G3				*p*-Values
Region	Deionized Water	Coffee	Cola	
Incisal	2.12 ± 0.28 ^a,A^	0.36 ± 0.10 ^a,A,B^	2.55 ± 0.55 ^a,B^	0.001 *
Middle	1.51 ± 0.33 ^b,A^	6.11 ± 0.33 ^a,A,B^	1.46 ± 0.42 ^b,B^	0.001 *
Cervical	6.65 ± 0.49 ^a,b,A,B^	5.04 ± 0.08 ^a,A^	5.21 ± 0.34 ^a,b,B^	0.003 *
*p*-values	0.001 *	0.001 *	0.001 *	
**G4**				*p*-Values
Region	Deionized Water	Coffee	Cola	
Incisal	17.15 ± 1.53 ^A^	5.18 ± 0.55 ^A^	0.59 ± 0.08 ^A^	0.001 *
Middle	10.43 ± 1.10 ^A^	11.11 ± 0.73 ^B^	3.63 ± 0.19 ^A,B^	0.001 *
Cervical	6.78 ± 1.10	11.02 ± 2.20 ^A^	5.60 ± 2.47 ^A^	0.037 *
*p*-values	0.001 *	0.003 *	0.015 *	

* Statistically significant at 0.05, small letters (^a,b^) showing significant difference within the solution, and capital letters (^A,B^) showing statistical significance between the solutions horizontally.

**Table 4:** Comparison between solution type and groups within each tooth region.

Groups	Incisal Region			*p*-Values
	Deionized Water	Coffee	Cola	
CG	10.45 ± 0.69 ^A^	11.71 ± 1.87	5.76 ± 1.38 ^a,b,A^	0.005 *
G1	5.88 ± 0.25	2.79 ± 0.72 ^a,b,c^	7.88 ± 0.67 ^a^	0.001 *
G2	2.43 ± 0.15 ^a^	4.29 ± 2.72 ^a,d,e^	4.74 ± 0.84 ^b,c^	0.269
G3	2.12 ± 0.28 ^a,A^	0.36 ± 0.10 ^b,d^	2.55 ± 0.55 ^c,d,A^	0.001 *
G4	17.15 ± 1.53	5.18 ± 0.55 ^c,e^	0.59 ± 0.08 ^d^	0.001 *
*p*-values	0.001 *	0.001 *	0.001 *	
	Middle Region			*p*-values
	Deionized Water	Coffee	Cola	
CG	12.66 ± 3.09 a,^A^	16.37 ± 1.66 ^a^	4.12 ± 0.97 ^a,b,c,d,A^	0.001 *
G1	2.87 ± 1.64 ^b,c^	6.87 ± 0.87 ^b,c^	5.09 ± 2.43 ^a,e,f^	0.083
G2	7.35 ± 0.55 ^b,d,A^	13.47 ± 2.37 ^a,d^	5.78 ± 1.40 ^b,e,A^	0.001 *
G3	1.51 ± 0.33 ^c,A^	6.11 ± 0.33 ^b^	1.46 ± 0.42 ^c,f,A^	0.001 *
G4	10.43 ± 1.10 ^a,d,A^	11.11 ± 0.73 ^c,d,A^	3.63 ± 0.19 ^d^	0.001 *
*p*-values	0.001 *	0.001 *	0.024 *	
	Cervical Region			*p*-values
	Deionized Water	Coffee	Cola	
CG	11.98 ± 2.01 ^A^	13.52 ± 1.47 ^A^	4.40 ± 0.46	0.001
G1	15.84 ± 1.21 ^A^	14.48 ± 2.34 ^A^	4.76 ± 0.40	0.003
G2	3.53 ± 1.14 ^a,b,A^	16.64 ± 1.68	7.07 ± 2.38 ^A^	0.001
G3	6.65 ± 0.49 ^a,c^	5.04 ± 0.08 ^A^	5.21 ± 0.34 ^A^	0.003
G4	6.78 ± 1.10 ^b,A,B^	11.02 ± 2.20 ^A^	5.60 ± 2.47 ^B^	0.037
*p*-values	0.001 *	0.001 *	0.333 *	

* Statistically significant at 0.05, small letters (^a,b,c,d,e,f^) showing non-significant difference within the solution, and capital letters (^A,B^) showing statistical non-significance between the solutions horizontally.

## Data Availability

The data will be available on request.
